# COVID-19 triggered a physically active lifestyle of people with cardiovascular diseases: Results of a small Austrian qualitative study

**DOI:** 10.3389/fpubh.2022.947250

**Published:** 2022-08-12

**Authors:** Eva Krczal, Walter Hyll

**Affiliations:** Department for Economy and Health, University for Continuing Education Krems, Krems, Austria

**Keywords:** physical activity (exercise), cardiovascular diseases, COVID-19, lockdown, coping strategies, outdoor exercise, intrinsic motivation

## Abstract

**Objective:**

This paper explores physical activity patterns and compensation strategies of people with cardiovascular diseases. The aim is to provide insights into the factors and their relationships that may affect physical activity levels positively or negatively during the pandemic.

**Methods:**

We adopted a qualitative approach with 35 participants who were purposively sampled from different provinces in Austria, including rural and urban areas. Semi-structured interviews were conducted during the second COVID-19 wave in autumn/winter 2020 and the fourth wave in autumn/winter 2021. Content analysis was applied to explore physical activity patterns, the perceived impact of the pandemic on physical activity as well as strategies adopted by participants to maintain physically active during the pandemic waves.

**Results:**

Results show encouraging signs of a recovery or even increase in physical activity during the pandemic waves. The main drivers for maintaining or even increasing physical activity were intrinsic motivation and self-determined motivation relating to the pursue of individual health goals. Furthermore, analysis suggests a reinforcing effect of exercising in green natural areas by decreasing perception of effort and increasing motivation. There was also one group who experienced difficulties in adapting physical activity behaviors. Study participants who were used to exercise indoors struggled to replace accustomed activity patterns with alternatives that were not impacted by lockdown restrictions.

**Conclusions:**

This study provides novel qualitative evidence on the effect of COVID-19 lockdowns on physical activity patterns of people with cardiovascular diseases. Public health interventions to enhance a physically active lifestyle during and beyond the COVID-19 pandemic are recommended to target moderate outdoor exercising and enhance adaptive capacities of people with cardiovascular diseases.

## Introduction

Since the onset of the SARS-CoV-2 pandemic, first reported in December 2019, political decision makers worldwide have adopted unprecedented measures to limit people's exposure to the virus and contain the spread of the disease (1). On the one hand, Public Health measures like social distancing, staying at home and the closing of cultural and sports facilities had been adopted to break the transmission of the virus. On the other hand, the same type of interventions negatively affected people's mental and physical health. During the lockdown phases, the population has developed a lifestyle characterized by lack of mobility, unhealthy diet, increased depression, loneliness, and psychological distress (2–6). Recent reviews indicate a decrease in physical activity and an increase in sedentary behavior across different populations during the lockdowns (7–9). It is not surprising that people who exercise in their free time are less likely to do so, for example, because of the closure of gyms. Limited physical activity, however, has the potential to increase the risk of many severe and disabling disorders (10). A reduction in physical activity and modified eating habits increase insulin resistance, total body fat, abdominal fat, and inflammatory cytokines, which in turn increase the risk of multiple chronic disease (11).

This paper addresses the question, how lockdowns affected physical activity patterns of people who need to exercise for health reasons, namely cardiovascular disease (CVD) patients. Physical inactivity represents an established risk factor for developing cardiovascular diseases and may have detrimental effects on those already affected (12–16). For CVD patients it is therefore essential for their disease management to practice physical activity on a regular basis. Since preexisting cardiovascular diseases increase the risk of experiencing severe COVID-19 complications (17), CVD patients represent a highly vulnerable group in consideration of the pandemic. Consequently, they were advised to take extra social-distancing measures. Vulnerable individuals were expected to be highly cautious during the pandemic to avoid a potential contact with the virus, thus limiting their opportunities for physical activities further. In addition, assuming that contacts to their physician or physiotherapist declined, the constant monitoring of adherence of stipulated exercise programs was interrupted.

Few studies exist, that focus on the impact of the pandemic on CVD patients' physical activity levels during the first *lockdown* waves. These studies show a decline in physical activity of CVD patients (18–23). There is one study suggesting that most study participants maintained or even increased their normal physical intensity during the first lockdown in UK (24). This study included stroke and heart disease patients in the sample. Yet, there is scarce evidence on the physical activity patterns of CVD patients over a longer time span including the *post-lockdown* period. One longitudinal study assessed changes in physical activity and sedentary behavior in Dutch CVD patients across the various phases of the COVID-19 lockdown between April and July 2020 (25). According to the authors, even when COVID-19 lockdown restrictions were gradually lifted, physical activity levels did not significantly change. Also, a US study on the physical activity patterns of CVD patients from two large cities confirmed a decline in physical activity during the first lockdown in spring 2020, which did not fully recover until October 2020 (26).

In this study, we, focus on a longer time span including the second/fourth waves on the one hand and, point to a maintenance or even an improvement in the physical activity level of CVD patients on the other hand. In an exploratory approach people with CVD were interviewed about their health behavior and physical activity patterns. Interviews were conducted during the successive waves of the pandemic in autumn/winter 2020/21 and autumn/winter 2021/22. Most study participants reported that they were able to maintain or even improve their physical activity level during the waves of the pandemic. These findings raise interest in the factors that influence physical activity patterns and adaptive capacities. We contribute to the existing literature in several ways. First, we provide additional evidence on the effects of a lockdown on physical activity of CVD patients up to January 2022. Second, the study is pointing to a recovery of physical activity levels during the course of the pandemic. Third, we outline determinants that may influence physical activity patterns as well as compensation strategies to restore physical activity levels throughout the pandemic.

A deeper understanding of the motives and experiences of people with CVD, when maintaining or adjusting their physical activity patterns throughout the pandemic, contributes to designing adequate public health interventions. These are necessary to enhance a physically active lifestyle during and beyond the COVID-19 pandemic.

## Materials and methods

### Data collection

The present research was part of a qualitative study exploring participants' perceptions on the impact of the pandemic on their lifestyles as well as their strategies to manage their disease and maintain a healthy lifestyle throughout the pandemic. During the interviews, physical activity emerged as a major concern for study participants. Therefore, this paper focuses on the physical activity patterns of study participants, the perceived impact of the pandemic on physical activity as well as strategies to maintain physical activity during the pandemic waves. A qualitative approach was adopted to explore perceptions, challenges and experiences from the perspective of participants and to provide insights into the complex relationships that may cause an increase or decrease of physical activity during the pandemic (27).

Selection criteria were set in advance. Participants were chosen along three dimensions: gender, age, and residence (urban or rural areas). The criteria are depicted in Table 1. The aim was to find a participant for each combination, which was almost achieved. Eligible participants were persons 40 years or over with cardiovascular diseases [diagnosed with heart failure or coronary heart disease, received bypass surgery, stent or cardioverter-defibrillator (ICD) implantation]. Exclusion criteria were incapacity to give informed consent and incapacity to articulate answers to interview questions without the help of others. The sampling strategy relied on a combination of convenience and purposive sampling (28). The initial strategy was to recruit participants by distributing an invitation to participate at the interviews at general practitioners' or specialists' practices and self-help groups in the field of heart failure. This strategy did not produce any response from eligible persons. Therefore, participants were recruited using existing personal contacts by research assistants working in health and social care. That is, participants stem from conveniently available sources. All persons, who were addressed personally, agreed to participate in the study and attended the interviews. Semi-structured interviews were conducted with 35 persons, who received health services in different provinces of Austria and from different care providers. Further, study participants differed in their perceived impairment of physical performance due to their disease or other comorbidities. As outlined above, purposive sampling was applied to account for diversity among study participants, to obtain relevant and diversified data relating to the research topic and to mitigate challenges of limited representativeness resulting from a convenience sample (28). Interviews were conducted by mutual agreement between interviewer and interviewee face-to-face in compliance with the applicable COVID-19 safety regulations or via zoom. Study participants were recruited during the pandemic waves in autumn/winter 2020/21 and 2021/22. Eleven interviews were conducted during the second wave between 2020-11-24 and 2021-02-05, another 24 interviews took place between 2021-10-27 and 2022-01-10 during the so-called “lockdowns light” in Austria. The study has been approved by the Ethics Commission of the University for Continuing Education, Krems (No. EK GZ17/2021-2024).

**Table 1:** Selection matrix.

	**Age**				
	**40–49**	**50–59**	**60–69**	**70–79**	**80^+^**
**Residence**					
Big city (100,000^+^ residents)		f VK3	f JC3 m KS1 m KS3 m KS4 m PJ3	m HB2 m HB3 f JC1 f PP2 m PP1 m PP3	f HB1 m PP4
Small town (3,000–99,000 residents)		f JC4 m KS2 m PJ2	f VK1		m JC2
Rural area (<3,000 residents)	f EL2 f EL3	f EL1 m EL6 f EL7 m EL9 f EL11	m HB4 f EL10 m PJ4 m VK2	m EL4 m EL5 m EL8 m VK4	f PJ1

f, female; m, male. Eligibility criteria: age 40+, cardiovascular disease diagnosed before the pandemic, heart failure or coronary heart disease or bypass surgery, stent or cardioverter-defibrillator (ICD) implantation. Exclusion criteria: incapacity to give informed consent, incapacity to articulate answers to interview questions without the help of others.

Interviews followed an interview schedule asking pre-defined questions, whereby modifications of questions or the introduction of new ones made room for unexpected and promising themes that emerged during the interviews. The interview schedule is provided in the Supplementary Table 1. At the end of the interview, socio-demographic data of study participants were collected with a short questionnaire. For a better contextualization of the physical activity patterns, study participants were asked to rate their perceived physical impairment due to the perceived burden of disease or other comorbidities following the New York Heart Association (NYHA) Classification (29).

Interviews were audio-recorded, anonymized and transcribed verbatim with annotations, which accounted for pauses, intonations, and nonverbal expressions of study participants to enrich answers with non-verbal information (30). All study participants provided written informed consent.

### Analysis

Content analysis was applied to produce a systematic and comprehensive overview of the data set based on participants' statements concerning physical activity patterns, the influence of the pandemic on them, and experiences when maintaining physical activity during the pandemic (31). Content analysis was used to explore, for example, how often certain strategies were mentioned and what these strategies were (31). Specifically, interviews were analyzed according to the content structuring qualitative content analysis (32). This method follows a structured process for arranging and analyzing interview data. The analytic procedure started with getting familiarized with the data by reading through transcripts, highlighting important text segments, writing memos, and case summaries. One third of transcripts was analyzed in this initial step by two researchers separately. Following initial text analysis, thematic main categories were developed, which formed the basis for structuring interview data. Three main categories were defined deductively based on the research question (“*Physical activity patterns*,” “*Impact of the pandemic on physical activity patterns*,” “*Strategies to maintain physical activity*”). In addition, another two main categories inductively derived from themes that emerged from the transcripts (“*Motivation for physical activity*,” “*Influencing factors*”). After the main categories and their descriptions have been developed, all data were coded along the main categories by two researchers independently. Coding of data involved assigning meaningful text segments to correspondent thematic main categories. Whenever text passages included different themes, multiple coding was applied (32). Coding of transcripts was aided by the qualitative data analysis software MAXQDA version 20 (www.maxqda.de/). After all data were coded along the main categories, sub-categories were inductively developed for a more refined structuring of the data set. The development of sub-categories involved compiling all text segments coded within the same main category to get an overview of relevant themes. Sub-categories were then defined according to central topics that emerged from the text. For the further course of analysis, definitions for sub-categories were formulated and supplemented with sample quotes. Next, all transcripts were coded once again along sub-categories. In an iterative process, coding was refined through review and further analysis. This procedure involved the creation of new sub-codes or the re-evaluation and re-definition of existing sub-codes. For example, Organismic Integration Theory (33), a sub-theory of Self-Determination Theory (SDT), emerged as a promising theoretical background to explain participants' motivation to maintain physical activity. Therefore, sub-codes on motivational factors have been reorganized following the Organismic Integration Theory.

Finally, the coded text segments were analyzed along the main categories to reach an overview of themes that emerged from the strategies and experiences of study participants regarding physical activity during the pandemic. In addition, possible connections between main categories and sub-categories were analyzed to explore complex relationships between main themes emerging from the data.

## Results

### Sample characteristics

Socio-demographic data of study participants are presented in Table 2. Study participants were living on average about 11 years (mean 11.06, *SD* 9.45) with their disease. Three participants were living with heart failure since birth or early childhood (not included in calculation of mean years of disease). Most study participants were male (63%), between 60 and 79 years old (56%), and lived in a partnership (83%). During the interviews, study participants indicated that they were embedded in a social network receiving support from their partner, family members, or friends during the pandemic. Analyses of transcripts revealed that participants displayed a good self-management of their disease (measuring blood pressure and weight daily or at least weakly, regular medical check-ups as advised by their physician or specialist, awareness of the importance of physical activity, and nutritional intake). Study participants mentioned that they attended regular physical therapy before the lockdowns (HB1, HB3, JC1), others outlined that they received exercise education during their rehabilitation stays before the pandemic (EL5, HB3, PJ1, PH4). Another group noted that they have learned physical exercises from regular attending group courses or a personal trainer (EL6, EL7, PJ3).

**Table 2:** Participant characteristics.

**Characteristic**	**Categories**	***N* = 35**
Gender	Female	13 (37%)
	Male	22 (63%)
Age	40–49	2 (6%)
	50–59	9 (26%)
	60–69	10 (28%)
	70–79	10 (28%)
	80+	4 (12%)
Employment status	Employed	9 (26%)
	Partial retirement	2 (6%)
	Retired	24 (68%)
Relationship status	Single	6 (17%)
	Partnership	29 (83%)
Residence	Big city	14 (40%)
	Small Town	5 (14%)
	Rural area	16 (46%)
relationship status	Single	6 (17%)
	Partnership	29 (83%)
Perceived physical impairment (NYHA-Classification)	I (none)	7 (20%)
	II (mild)	7 (20%)
	III (moderate)	15 (43%)
	IV (severe)	6 (17%)

### Main categories

Qualitative content analysis yielded five main categories: “*Motivation for physical activity*,” “*Physical activity patterns*,” “*Impact of the pandemic on physical activity patterns*,” “*Strategies to maintain physical activity*,” and “*Influencing factors*.” Table 3 displays these main categories, sub-categories, and the number of corresponding quotes. For example, seven quotes refer to intrinsic motivation as a driver for participating in physical activity. A description of main and sub-categories as well as sample quotes are provided in Supplementary Table 2.

**Table 3:** Main codes, sub-codes, and frequency of physical activity patterns during the pandemic.

**Main codes and sub-codes**	**Frequency**
**1. Motivation for physical activity**	**52**
1.1 Intrinsic motivation 1.2 Integrated regulation 1.3 Identified regulation 1.4 Introjected regulation 1.5 External regulation 1.6 Amotivation	7 6 32 1 2 4
**2. Physical activity patterns**	**47**
2.1 Moderate outdoor exercise 2.2 Moderate to vigorous indoor exercise 2.3 Moderate to vigorous exercising at home 2.4 Leisure behavior	11 10 7 19
**3. Impact of the pandemic on physical activity patterns**	**93**
3.1 Continuation of moderate physical activity with no restrictions 3.2 Cessation/reduction of moderate to vigorous physical exercise 3.3 Continuation of leisure behavior with no/minor restrictions 3.4 Cessation/reduction/modification of leisure behavior	13 20 18 42
**4. Strategies to maintain physical activity**	**80**
4.1 Integration of walking into daily routine 4.2 Integration of moderate exercising at home into daily routine 4.3 Increase in outdoor leisure activities 4.4 Increase in moderate outdoor exercise 4.5 Introduction/increase of moderate to vigorous outdoor exercise 4.6 Replacement indoor exercise by moderate outdoor exercise 4.7 Replacement indoor exercise by moderate to vigorous exercise 4.8 Resuming physical exercise due to release of measures	12 25 19 4 2 8 3 8
**5. Influencing factors**	**55**
5.1 Physical impairment 5.2 Time of the year 5.3 Availability of time 5.4 Partner, friends 5.5 Availability of garden/proximity to green natural areas	22 5 9 7 12

### Motivation for physical activity

Analysis of transcripts revealed that participants were motivated for engaging in physical activity mostly for self-determined reasons. Engagement in physical activity to achieve desired health outcomes emerged clearly as the most frequently cited and emphasized motive among study participants. This attitude relates to “*Identified regulation*,” a moderate form of self-determined external motivation (34). Being physically active was believed to be an essential part of participants' disease-management supporting them to remain healthy. In particular, regular practice was considered important to strengthen the heart muscle or to boost the immune system. Other motives for engaging in physical activity related to a change in lifestyle after a heart attack or to strengthen physical fitness after an operation. For some study participants, being physically active was considered a promising strategy to cope with the pandemic:

“*And I'm sure if I hadn't done that before, I wouldn't have survived. So, if I had just been sitting around and stuff.” [HB2: 50–50]*

Next, analysis of transcripts revealed expressions relating to “*Intrinsic motivation”* (31) among certain study participants. Being physically active was associated with feelings of pleasure, enjoyment, and fun. One participant explained:

“*And that did me a lot of good, because you feel better afterwards.” [EL2: 103–103]*

Some study participants indicated that they have incorporated physical activity in their personal value and belief system by emphasizing that they considered themselves as physically active persons. Motives relate to the “*Integrated regulation”* dimension, the most self-determined form of external motivation (34). One participant claimed:

“*I am physically even better off than the so-called healthy people, because I have done sports every day. Always. Before, after, and during Corona.” [KS3: 10–10]*

It appeared that only a few study participants were physically active for extraneous motives. Two study participants made a comment that their physician advised them to exercise regularly, which belongs to the “*External regulation”* dimension of external motivation (34).

In summary, it was apparent that most study participants were motivated to be physically active for self-determined reasons.

### Physical activity patterns

Different physical activity patterns were driven by study participants' individual preferences and perceived physical impairments. Table 4 provides descriptions and examples of physical activity patterns that emerged from the analysis. One group preferred moderate outdoor exercise, for example hiking or biking. Another group favored moderate to vigorous indoor exercise, such as training in a fitness club. Other study participants reported moderate to vigorous exercising at home.

**Table 4:** Description and examples of physical activity patterns derived from content analysis.

**Physical activity patterns**	**Description and examples**
Moderate outdoor exercise	Moderate forms of outdoor exercise: powerwalking, hiking, biking, golf, archery, skiing
Moderate to vigorous indoor exercise	Moderate to vigorous forms of exercise using indoor sport facilities: group fitness, exercising in a fitness club, swimming, team sport, aerobics
Moderate to vigorous exercising at home	Moderate to vigorous forms of exercising at home: gymnastics at home, TV-led exercise programs, Cardio-training at home
Leisure behavior	Walking and other leisure behavior: going shopping, housekeeping, gardening

For some participants, going out for a walk represented an essential component of their physical activity program. Furthermore, some participants considered leisure behavior like going shopping, doing housework, or gardening as vital part of their daily physical activities.

From the text analysis it became apparent that the reported intensity and duration of physical exercise was subject to individual framing and perceived physical impairment. For example, exercising 30 min gymnastics in the morning was considered either the prime component of daily exercising for one study participant with major physical restrictions or one element, among other daily activities, for another study participant with minor restrictions.

### Impact of the pandemic on physical activity patterns

Insights gained from the analysis showed that study participants experienced the impact of the pandemic on their physical activity patterns in different ways. Those study participants who were used to exercise outdoors stated that they didn't feel much difference during the pandemic because they had the same possibilities to be physically active compared to pre-COVID-19 times. One study participant explained:

“*I don't go to fitness studios anyway. I move in the great outdoors. I go for walks and hikes.” [PJ4: 37–37]*

Another study participant pointed out that he used to exercise with a private personal trainer, and they continued training throughout the pandemic. Physiotherapy and rehabilitation on the contrary, were reported to be canceled at least during the first lockdown.

Also, those study participants, who were used to practice regularly at home, continued their exercise as usual. One study participant emphasized:

“*Exercises and I do all that myself... I did all that and I still do it exactly the same today.” [PJ1: 69–71]*

Gardening or hobbies in green natural areas like collecting mushrooms in the forest represent typical leisure activities, which were not perceived to be affected by lockdown restrictions:

“*And that [gardening] was actually a continuous activity that has remained. Nothing has changed.” [KS4: 132–132]*

Another theme, that emerged from the interviews, was the cessation or reduction of moderate to vigorous indoor exercise. Due to lockdown restrictions, public sport facilities and fitness clubs had to close several times depending on current infection rates. Even when fitness clubs or rehabilitative facilities reopened after the lockdowns, there was some reluctance to resume exercising, because certain study participants, considering themselves as high-risk persons, preferred to limit potential contact with the virus.

In contrast, outdoor sporting facilities were allowed to reopen soon after the first lockdown in March 2020. Study participants who, for example, were used to practice golf could resume their physical activities after a few weeks. Another study participant mentioned that she could resume individual physiotherapy after 6 weeks following the announcement of the first lockdown.

Finally, the impact of the pandemic situation on leisure activities, that involved walking, was discussed during the interviews. The continuation or reduction of routine activities like going shopping was perceived to impact daily steps. Especially, study participants, who were used to go out frequently for entertainment, experienced radical changes in leisure activities with the closing of restaurants, pubs and cultural events. Consequently, they reported a reduction of daily movements. One study participant reflected:

“*One becomes somehow, something like pensionistic.... you don't move out, you don't have the social, active life. The exhibitions were all canceled, all the activities, going abroad and so on, that was all gone. My whole leisure life was completely stopped.” [VK2: 59-59]*

In summary, experiences on the impact of the lockdown restrictions related to accustomed physical activity patterns. Study participants, who were used to indoor sport and going out for entertainment, appeared to be discouraged by continued lockdown restrictions, while study participants, who were used doing outdoor exercises or who possessed a garden, claimed to be hardly affected by restrictions. Some individuals from this group even expressed positive associations with the pandemic situation, which offered to them opportunities for relaxation, recreation, and creativity.

### Strategies to maintain physical activity

Study participants adopted different approaches aiming to remain active during the pandemic. One strategy that emerged from the transcripts was the integration of regular exercise into daily routine, thus increasing the frequency (and duration) of physical activity. Going out for a walk and walking instead of using public transport became a preferred mode for leaving home, getting out, and practicing physical activity.

“*And just go for a walk a lot.... we've really been walking since Corona, now the second lockdown, three, four kilometers every day. Even when it is already dark, we go our rounds.” [EL1: 47–47]*

“*And I walked five or six kilometers every day, in an hour or an hour and a half. That's actually how it all started. Although I have actually walked before, powerwalking you have to say, I just walked more.” [EL11: 3–3]*

Next, study participants engaged in regular exercising at home using home training equipment or performing exercises they have learned from their therapist or from group training:

“*Because I have had a lot to do with physiotherapists in the last three years. And I've actually picked up quite a lot from them that I can use for myself when I have a pain. In addition, I have always been pretty well briefed by the sports union, (…) which you can actually perform yourself. And a lot has been actually stuck.” [EL7: 49–49]*

Other study participants attended TV or online-training programs. However, it appeared from the transcripts that exercising at home was considered a poor alternative for group training, because study participants missed the group feeling:

“*And the first shock is then again: ah, now we can't go again. And then you try to do something at home. (...) You already do your exercises, but the key is always to go somewhere and exercise together in a group.” [EL6: 117–117]*

“*During the lockdowns, the fitness clubs also offered online-Zumba and then you jump around in front of your laptop in the kitchen and feel relatively stupid. The main problem is that these sports have to be fun, otherwise I don't do them permanently. For example, Zumba depends a lot on having a nice group and a good trainer. And of course, the group experience is gone when I'm jumping around in front of the computer. No, the motivation is much more difficult.” [VK3: 61–6]*.

Another strategy that emerged was the replacement of indoor activities by outdoor activities. The most favorite outdoor activities were moderate activities like hiking and biking.

“*It was actually a compensation. What would you have done? As I said, you used to go to the gym, two or three times. It was actually the morning occupation. And then I just went out for a walk almost every day.” [HB4: 32–32]*

Fewer participants engaged in moderate to vigorous outdoor exercising. One participant reported regular training in an outdoor parcour, another one reported to have intensified regular outdoor cardio training. Other replacement strategies included swimming in the lake instead of using the public swimming pool or chopping wood instead of training in the gym.

In summary, there were encouraging signs amongst most study participants to adopt a proactive approach in maintaining physical exercising during the pandemic waves.

### Influencing factors

Transcripts contained descriptions of influencing factors which acted either as facilitators or barriers to physical activity.

The availability of a garden or the proximity to green natural areas facilitated engagement in outdoor activities. In fact, the availability of a garden emerged as a welcome opportunity to be physically active in a private and safe environment:

“*Well, because of the garden, the possibility to move and to do something useful is quite big … I didn't do anything in a gym before and I didn't do anything after, so this is all happening at home. There have been no restrictions at all.” [KS1: 46–46]*

Also living close to green natural areas encouraged study participants to be physically active.

“*And actually, I started walking and I didn't go into the town or anything like that, only out in the fields and into the meadows.” [EL11: 3–3]*

For some study participants the time of the year played an important role for their motivation to exercise:

“*At the first lockdown, you have to remember that the weather was different. You had beautiful days there. Those days have lasted longer. (...) Everyone was looking forward to being outside. Many people went outside. Now, the second lockdown is like this: at four o'clock it's dark. And in my opinion, people will become more depressed now. Because it's dark, it's cold.” [EL2: 11–11]*

Availability of time emerged as enabler for practicing physical activity. Many study participants were already retired. They had the timely resources to organize their daily activities. Others had more time available because they were classified as high-risk persons and could stay at home for sickness leave. A few participants noted that they had home office arrangements with reduced working times. Home office arrangements allowed a better time management for integrating physical exercises into daily routines.

The social network (partner, family, friends) appeared to act in both ways, either encouraging or limiting physical activity. The accompaniment of a partner, friends, or a dog seemed to motivate study participants to engage in regular exercising. On the other hand, a partner who is not willing or able to practice may reduce physical activity. Study participants outlined that family members or partners did the shopping. On the one hand, this was a measure to limit possible exposure to the virus, on the other hand, it was reducing daily steps.

### Cross-category analysis

Analysis of main categories is supplemented by an exploration of the relationships between categories to provide a more contextualized and comprehensive picture of the impact of the pandemic on physical activity patterns. Cross-category analysis revealed interconnections and interactions between main categories in different ways. Apart from the connection between “*Physical activity patterns*” and “*Impact of the pandemic on physical activity patterns*” the most obvious connections that emerged from analysis are presented in this section.

For a better contextualization of those relationships, study participants were grouped into three categories: One group reporting a perceived increase in physical activity during lockdowns, a second group claiming to have maintained their level of physical activity, and a third group who felt that their physical activity had decreased. We exclude seven participants from this analysis, because, due to external factors, an adjustment of the activities was not at their discretion. One participant reported an increase in physical activity due to his recent rehabilitation stay. Five participants reported a decrease in physical activity due to an injury, comorbidity, or aggravation of disease. Another participant made the point that his physical activity was not impacted by the pandemic because he was never much engaged in physical activity before, during and after the pandemic. Table 5 summarizes changes in physical activity patterns as well as motivational factors indicated by participants.

**Table 5:** Changes in physical activity and motivational factors.

	**Motivational factors**				***n* = 28**
	**Intrinic motivation**	**Integrated regulation**	**Identified regulation**	**Not reported**	
**Changes in physical activity patterns**					
**Increase in physical activity**					**6 (21%)**
Increase in walking	1		1		2 (7%)
Increase in outdoor exercising	2		2		4 (14%)
**No changes**					**14 (50%)**
Continued as usual			4	3	7 (25%)
Indoor exercising replaced by outdoor exercising	1	4	2		7 (25%)
**Reduction of physical activity**					**8 (29%)**
Regular exercising at home but less walking			3		3 (11%)
Less indoor exercising			1	3	4 (14%)
Less walking				1	1 (4%)

Percent refer to total number of participants.

### Cross-category analysis between “Motivational factors” and “Strategies to maintain physical activity”

The first group represents six study participants (21%) who claimed to have increased their physical activity by increasing frequency and duration of walking and/or moderate outdoor exercises (see Table 5). These individuals pointed out that they were already engaged in walking and moderate outdoor activities before the pandemic and continued or even increased exercising throughout the pandemic.

“*I just look forward to walking every day in the morning or afternoon, for me personally (...), I have to say that I have been moving more, quite simply. And that I just enjoy it.” [EL11: 233–233]*

Similar connections have emerged from the analysis of the second group, composed of fourteen study participants (50%), indicating that they maintained their level of physical activity (see Table 5). Seven participants continued with outdoor leisure activities like walking or gardening and moderate exercising outdoors or at home, because these activity patterns were not affected by lockdown restrictions. The other seven participants replaced indoor exercising by longer and more frequent outdoor leisure activities (walking, gardening) and/or moderate to vigorous exercising outdoors or at home (for example, replacing group training by online-training at home). These individuals reported to be frequently engaged in different physical activities.

Insights from the analysis of the first and the second group revealed intrinsic motives, thus indicating a relationship between “*Motivational factors*” and “*Strategies to maintain physical activity*,” specifically between “*Intrinsic motivation*,” “*Integrated regulation*,” and “*Increase in outdoor leisure activities*” as well as “*Increase in moderate outdoor exercise*.”.

### Cross-category analysis between “Availability of garden/proximity to green natural areas” and “Impact of the pandemic on physical activity patterns”

Analysis of the second group revealed another interaction, namely between the influencing factor “*Availability of a garden/proximity to green natural areas*” and “*Impact of pandemic on physical activity patterns*.” Eleven participants (out of 14) from this group were living in rural areas or possessed a garden or a second home in rural areas. Study participants noted that having an own garden enabled them to pass lockdowns without major restrictions. Similarly, study participants living close to green natural areas noted that it was easy for them to continue or even increase outdoor activities:

“*With sport and with fresh air the first lockdown went pretty well.” [EL6: 7–7]*

The third group consists of eight study participants (29%) indicating that their level of physical activity has decreased (see Table 5). One group of three participants noted that they continued with regular moderate exercising but noticed a decrease in general movements (going shopping, going out). Two of those lived in an apartment in a big city. Another group of four study participants indicated a reduction in physical activity due to a decrease in moderate to vigorous exercising. These were all individuals accustomed to indoor training and explained that they did not find adequate alternatives during the lockdowns. Three of them were living in urban areas, two in an apartment. Finally, one person mentioned a reduction of general movements (less going out) until vaccination was received. Also, this participant lived in a big city.

Insights from the third group reveal that “*Moderate to vigorous indoor exercise”* and “*Availability of garden/proximity to green natural areas*” was highly connected to “*Cessation/reduction of moderate to vigorous physical exercise*.” Participants who lived in urban areas and were used to exercise indoors struggled to find adequate outdoor alternatives during the lockdowns:

“*I did much less sport, because apart from going for a walk you couldn't do anything at the beginning and I don't like going for a walk very much, I'm more of a swimmer or fitness club exerciser, Zumba, and we finally brought ourselves to do something, and we went out for a walk or a small bike ride almost every day.” [VK3: 9–9]*

For these participants the closure of sport facilities appeared to produce feelings of frustration or helplessness:

“*Before Corona I went swimming regularly and then that was no longer possible, because now of course I couldn't go through with it. I just slacked off and now I'm still waiting. Until they unlock.” [JC3: 58–58]*

In the further course of the pandemic waves in autumn/winter 2020 and 2021 this frustration was nurtured by insecurity deriving from continued closings and reopening's:

“*Everything was then actually over from March 15 and of course also the uncertainty, who is still open then and what do you really get then, so the uncertainty and the disappointment together.” [KS4: 20–20]*

The relation to motivational factors remains unclear in this group. Four individuals (out of eight) did not emphasize their motivation for physical exercise during the interview, the other four, among them those who continued regular exercising at home, expressed motives relating to Identified regulation (see Table 5).

## Discussion and limitations

This paper explored how the pandemic waves in 2020 and 2021 affected physical activity patterns of people with CVD. Previous research outlined a general trend of reduced physical activity due to COVID-19 lockdown restrictions for CVD patients as well as other populations. This evidence is related to the effect of the first lockdown in spring 2020. Findings of Vetrovsky et al. suggest a 16% decrease in step counts of heart failure patients during COVID-19 quarantine (18). Compared to the present study, they focus on a short time period, namely on the first 3 weeks after the first quarantine in the Czech republic. Another study by Fagih et al. also reported a significant decline of 27% in physical activity during the lockdown periods between February and April 2020 due to the pandemic (19). Similar results have been shown by Sassone et al., who analyzed how the first forced 40 day in-home confinement in Italy affected physical activity of patients with automatic implantable cardioverter-defibrillators. They find a 25% decline in physical activity after the lockdown began (20). Due to the short time span long run effects are not mapped.

Our results point out that behavior might change again in the long run such that physical activity levels recover. When analyzing transcripts, it became apparent that during and after the first pandemic wave, most study participants developed coping strategies and were able to adapt to pandemic circumstances. Findings of this study display encouraging signs pointing to a recovery of physical activity over the long run. Most participants in our study (71%) reported that they managed to maintain or even increase pre-COVID-19 physical activity levels. Specifically, 21% of study participants claimed to have increased physical activity and 50% of participants indicated that they have maintained their level of physical activity.

Our findings are supported by emerging evidence from longitudinal studies, suggesting a gradual recovery of physical activity levels following the time span after the first lockdown. In line with previous research, Lu et al. reported that physical activity has reduced among patients with pre-existing cardiac diseases during the first lockdown (26). Their findings further suggest that physical activity decreased the most during the first 3 weeks of the emergency quarantine order, and then started to slowly increase. Yet, in their study patients did not return to pre-restrictions levels till early October 2020.

Findings from other studies suggest that certain populations were more successful in maintaining physical activity levels than others. Rogers et al. assessed the impact of the first lockdown on physical activity behavior of adults with serious health problems or self-perception of high risk from COVID-19 (24). In line with the results of our study, most participants (75%) maintained or even increased their normal physical activity intensity. One UK study outlined that older people were more likely to maintain and recover their physical activity levels compared to younger counterparts (35). These results are supported by a US nationwide Coping Study conducted during the first pandemic wave between April and May 2020 (36). Insights from this US study highlight the resilience of older adults to cope with adverse consequences of the pandemic. Our findings support the idea that certain populations, in this case individuals who are aware of the need to exercise for health reasons, are more resilient to the impact of the pandemic than others by developing adaptive skills to cope with the unique pandemic situation.

Our results clearly show that the most successful group (regarding coping strategies) were those who have integrated walking or moderate outdoor activities into daily routine. This is not surprising, because activities like walking, biking, or hiking were not affected by lockdown restrictions, as long as they were practiced alone, or with a person from the same household. The findings are supported by a study from New Zealand reporting that moderate active individuals were more likely to maintain or even increase the intensity of physical activity during and after the first lockdown (37).

It is important to note that previous research assessing the impact of the pandemic on physical activity of people with CVD is lacking information on factors that may affect a change in behavior. This study presents refined findings, providing a deeper understanding of the reasons why certain individuals were more successful in recovering physical activity than others.

For example, van Bakel et al. conducted an online questionnaire for patients with CVD to assess physical activity before and during the first lockdown restrictions (April 2020) in the Netherlands (22). They find that moderate-to-vigorous physical activity increased mainly due to an increase in time spent walking and doing odd jobs, while time spent exercising declined. There is no information, whether the decrease in exercising is the result of closure of sport facilities. In a follow up study van Bakel et al. show that overall moderate-to-vigorous physical activity did not change between April 2020 and July 2020 (25). The effect of reopening of sport facilities remains unclear.

Our findings suggest that adaptive capacities depend on existing physical activity patterns. This would be in line with the Theory of Planned Behavior (38). According to this theory, health behavior is determined by personal beliefs, attitudes, expectations, self-efficacy, and intentions of individuals. Thereby, people are more likely to adopt certain behaviors when they feel confident to be able to perform them (38). Insights from our analysis show that those participants, who were already engaged in outdoor activities or training at home prior to the pandemic, more easily compensated gym visits or group trainings by increasing outdoor or home exercising. That is, they were already used to perform those activities. In contrast, participants, who were used to exercise only indoors, struggled to adapt their physical activity routine during the pandemic. These findings suggest that they might lack confidence in engaging in novel types of physical activity or in exercising alone (39). This could be an explanation why certain participants did not find appropriate alternatives during the pandemic.

Our results further suggest that study participants favor consistency in physical activity patterns. The insecurity imposed by the COVID-19 specific context may pose an obstacle in the process of adapting activity behaviors. On the one hand, participants in our study outlined that they were waiting to the end of the pandemic (lasting longer than initially expected). On the other hand, analysis suggests that participants were frustrated by continued closings and reopenings of sport facilities. This implies that they might refrain from resuming indoor activities, that carry the risk of being restricted in the near future due to a newly upcoming pandemic wave.

Next, we found that the availability of a garden or the proximity to green natural areas emerged as a distinct factor shaping physical activity behaviors. More precisely, having an own garden supported participants to exercise in a private and safe environment. Also living close to green natural areas offered participants the possibility to be physically active in surroundings with a low perceived risk of exposure to the virus. Thus, possessing a garden or living in rural areas acts as an enabler for physical activity during the pandemic. Our findings are supported by the study of Rogers et al., who highlight the importance of access to green or open spaces for maintaining physical exercises during the pandemic (24). In a similar vein, Labib et al. suggest the exposure to the natural environment during the first 2 years of the COVID-19 pandemic inter alia improved physical activity (40). That nature helped individuals to cope with the pandemic and maintain health and wellbeing was also shown by Robinson et al. (41). Furthermore, our findings provide an explanation for the positive effect of living in rural areas observed in a study by of Chague et al. who interviewed congestive heart failure patients during the sixth and seventh weeks of the first lockdown in France (21). Over 40 percent had indicated a decrease in physical activity, whereas patients living in rural areas were less likely to decrease their physical activity (half as often) compared to urban populations.

To better understand how motivational drivers influence the level of physical activity, our analysis relied on Self-Determination Theory (SDT) which offers a conceptual basis for exploring physical exercise motivation (42, 43). Following SDT the motivation to participate and persist in physical activity may be self-determined (freely initiated by the individual) or externally controlled (through pressure from others) (33).

Our findings are consistent with previous research suggesting that individuals, who are more intrinsic and self-determined in their motivation, tend toward more frequent and regular physical exercise (44). Especially, *Intrinsic Motivation* and *Integrated Regulation*, the most self-determined form of external regulation, emerged as distinct motivational factors for increasing outdoor activities during lockdowns. In addition, cross-category analysis suggests a reinforcing effect of exercising in green and natural areas. Compared with exercising indoors, exercising outdoors results in greater feelings of revitalization, enjoyment, and satisfaction (45). Furthermore, a review by Gladewell et al. suggests that green exercise reduces perceived effort by enhancing mood and reducing awareness of physiological sensations, as well as negative emotions. This in turn might increase motivation (46).

Other studies referring to SDT in exploring the physical exercise behavior of CVD patients report self-determined motivation to be a significant predictor of long-term exercise behavior (47) as well as on exercise volume and length of exercise session duration (48). Consequently, physical activity programs are generally designed to facilitate more self-determined regulation of behavior, promote the fulfillment of basic needs, offer choice, and avoid external pressures for compliance (42).

In our sample, participating in physical activity was considered as a key component of maintaining a healthy lifestyle. Insights form the transcripts suggested that study participants felt confident in managing their disease and in exercising without the consultation of a trainer, therapist, or physician. Participants even reported that they had less personal contact to their physician or therapist during the lockdowns. On the one hand, the absence of external regulation (instructions by a therapist or physician) may have triggered study participants to take more responsibility for their own health and to improve their health behavior toward a healthier lifestyle. Consequently, they might be more self-determined in their motivation to exercise physical activity, which is suggested to enhance the frequency and persistence in exercising. In our sample, there were only two individuals indicating that they followed the advice of their physician in exercising. However, these study participants referred to pre-COVID-19 times when having received physician's recommendation for exercising.

On the other hand, there are reasons to assume that external regulation in the form of supervised training may play a decisive role for patients' motivation. If less experienced or physically impaired individuals need more assistance, the absence of a therapist or physician might be discouraging for this group. The positive effect of external regulation has been suggested by a study of Kulnik et al. (49). The study assessed the change in exercise capacity during the first COVID-19 lockdown in spring 2020. The study was conducted on patients in cardiac rehabilitation, that had been attending weekly supervised group-based training sessions prior to the first lockdown. Their findings suggest that exercise capacity reduced over time. Especially this group of patients was in favor of professional supervision of their training and of the motivational effect of training together. The authors explicitly “acknowledge the potential for selection bias, whereby study recruits might represent more exercise-conscious patients who were more motivated to return to group-based CR sessions after lockdown” (p. 10). Kulnik et al. further outline that CVD patients found alternatives for a supervised group training as training at home or outdoors, going for walks, or doing gardening. But, those activities did not compensate for the group training (49).

In line with the findings of Kulnik et al. our study shows a decrease in physical activity for certain individuals from the group who used to exercise indoors. Only four participants in our sample missed the trainer and the group feeling when exercising alone and found it harder to adapt to changing circumstances and switch to autonomous training.

The effect of external regulation and autonomous training on the persistence in physical activity in people with CVD may be worth being examined in future research, given the distinct situation and health needs of this vulnerable group.

The results of this study can be used for refining public health initiatives. Previous studies recommend home-based exercise (50) programs combined with supervision for high-risk patients (16) to promote the maintenance of physical activity of people with CVD and other vulnerable groups during a pandemic. Our results show that participants prefer easily accessible media like television for participating in home-based exercising. Further, our findings emphasize the benefits of moderate outdoor exercising which resulted in the highest consistency in physical activity patterns throughout the pandemic. This implies that physical activity programs for vulnerable populations should include a mix of indoor and outdoor exercising, so that people can choose between different alternatives in the event of a closing of sports facilities. Home-based exercise programs could be extended to instruct participants in how to use walking sticks, or how to plan and organize activities like hiking or biking. Furthermore, home-based exercise programs could prepare participants for subsequent outdoor activities, for example offering joint warm-up sessions and motivating participants to continue exercising outdoors. In addition, supervised outdoor group training programs could attract persons who seek professional advice and group feeling.

In our sample, the decrease in physical activity resulted from difficulties experienced by certain study participants to adapt physical activity behaviors. Study participants who were used to exercise indoors were confronted with a discontinuation of accustomed exercising habits which seemed to impact their physical activity levels even beyond the lockdown period. We therefore recommend lifestyle coaching programs promoting adaptive capacities of people and thereby help individuals to leave familiar exercising habits and find alternatives. Supporting people in developing coping strategies could strengthen their resilience in future distortions.

In support of previous research, our findings show that family encouragement may facilitate or hamper physical activity (51). Results suggest that study participants prefer companionship of a partner, friend, or a dog when exercising outdoors. Analysis revealed an unintended negative effect on physical activity resulting from children or partners offering support in doing the shopping. On the one hand, the prime motivation was to protect a high-risk person from a possible exposure to the virus. On the other hand, this was reducing their daily steps. Public health planners are recommended to re-evaluate measures like introducing special shopping hours reserved for elderly or vulnerable persons to avoid crowded supermarkets, which has been introduced as a general recommendation in Austria during the first lockdown.

Assuming that the general population behaves similar than our study participants, our findings can be used to promote physical activity for a wider target group. Previous studies highlighted time availability as an important facilitator or barrier to physical activity during the pandemic (52, 53). This was also reflected in our study findings. Study participants pointed to the importance of having enough time for integrating regular exercising into daily routine. Adapting working times or opening times of public childcare facilities to enable a better time management could support people to introduce regular exercise in their daily routine. Based on the finding that individuals, who were used to outdoor exercising, continued to be active, we recommend urban planners to incorporate health aspects and design easily accessible spaces for walking, cycling, and active living. In a similar vein, Levinger et al. highlight the benefits of outdoors for physical and mental health in general and point to the importance of better access to parks and nature in urban locations. Thereby, needs of the elderly population and people with disability must be taken into account (54). Possible incentives could be the placement of information signs and outdoor equipment promoting easy to follow physical exercises or providing walking, running, or biking tours in living areas.

There are some limitations which need to be considered when interpreting study results. First, our sample consists of individuals with advanced disease self-management capacities. The majority of study participants were retired. Those who were working indicated that they had more leisure time during the lockdowns. This special group had more time at their disposal to reflect and manage their lifestyle. Therefore, the results presented in this paper are limited regarding representativeness. Second, the study is designed as exploratory research aiming to detect possible determinants of physical activity during the pandemic. The results, especially from the sub-analyses with small sample sizes, suggest relations, which need to be investigated in quantitative studies using larger sample sizes. Further research could also focus on gender or age differences, which we could not consider due to the small sample size especially in the sub-analyses. Third, compared to other research examining the impact of the pandemic on the level of physical activities, this study did not apply objective measures to assess the level of physical activity like using accelerometer data. Therefore, findings might be affected by imprecise assessments of physical activity due to differing reference frames of study participants. However, it allowed participants to evaluate changes in physical activity within their own reference framework, which can hardly be considered in quantitative study designs. For example, in the study of Vetrovsky et al. all patients were participants of an ongoing randomized controlled trial of an outside walking intervention (18). Their outcome measure are step counts based on wearing an accelerometer. Since their results are based on outdoor activity and might simply reflect restrictions on outside activities, the authors cannot rule out that a substitution with indoor activities took place. One participant from our study for instance outlined that he started the day with morning gymnastics in the bed. Moreover, certain intercorrelations can hardly be considered in large quantitative studies. Five participants (14%) in our sample reported a decrease in physical activity due to an injury, comorbidity, or aggravation of disease. In this case, the reduction in physical activity was not directly linked with lockdown measures.

## Conclusion

Previous research on the impact of the pandemic on physical activity patterns of people with cardiovascular diseases pointed to a decline in physical activity levels during the first lock down phase. Consistent with recent longitudinal studies we show a recovery of physical activity levels under certain conditions. Findings suggest that participants, who were accustomed to moderate outdoor or home exercising, were able to maintain or even increase pre-pandemic physical activity levels by continuing or intensifying activities that were not impacted by public health restrictions. Only a few participants, who were used to indoor exercising prior to the pandemic, experienced difficulties in adapting their physical activity patterns to the pandemic situation. Furthermore, exercising in green natural areas turns out as multiplicator for engagement in physical activities presumably via motivation. Intrinsic motivation and self-determined motivation are shown to be main drivers for maintaining or even increasing physical activity. Results suggest that public health interventions should promote outdoor activities, design healthy cities with easily accessible green natural areas and provide lifestyle coaching to enhance adaptive capacities to be better prepared for a potential upcoming pandemic wave.

## Data availability statement

The datasets presented in this article are not readily available because transcripts may include confidental and personal information about study participants. The interview schedule, main and sub-categories, their descriptions as well as original contributions of study participants (sample quotes) are provided in the article/Supplementary material. Requests to access the datasets should be directed to EK, eva.krczal@donau-uni.ac.at.

## Ethics statement

The studies involving human participants were reviewed and approved by Ethics Committee of the University for Continuing Education Krems, Austria. The patients/participants provided their written informed consent to participate in this study.

## Author contributions

EK designed the study. EK and WH contributed to data interpretation. EK wrote the manuscript with support of WH. WH contributed to literature research, literature analysis, and focused on core messages and finetuning of the paper. Both authors have contributed to, read, and approved the submitted version of the manuscript.

## Conflict of interest

The authors declare that the research was conducted in the absence of any commercial or financial relationships that could be construed as a potential conflict of interest.

## Publisher's note

All claims expressed in this article are solely those of the authors and do not necessarily represent those of their affiliated organizations, or those of the publisher, the editors and the reviewers. Any product that may be evaluated in this article, or claim that may be made by its manufacturer, is not guaranteed or endorsed by the publisher.

## Abbreviations

CVD: Cardiovascular Diseases
SDT: Self-Determination Theory.
