# Supplementary material for: COVID-19 triggered a physically active lifestyle of people with cardiovascular diseases: Results of a small Austrian qualitative study
Source: Front Public Health. 2022 Aug 12;10:947250. doi: 10.3389/fpubh.2022.947250 (PMC9417466; doi:10.3389/fpubh.2022.947250)
Supplement: Supplementary file 1 [file Table_1.pdf]

Supplementary Table 1: Interview Schedule

**Main questions and sub-questions**
1. How did you experience the period since the start of the corona pandemic in March 2020?<br>• What particular experiences or highlights do you remember?<br>• How did you experience the first wave and the first lockdown in March 2020?<br>• How did you feel about the subsequent lockdowns in November/December 2020 and the "Light" lockdowns in spring 2021 compared to the 1st lockdown in March 2020?<br>• Is there an experience during the Corona pandemic that particularly stuck in your memory and could you describe it to me?
2. How important is contact with your primary care physician to you?<br>• How often do you have personal appointments or telephone consultations?<br>• How has the availability of your family doctor changed during the pandemic compared to before?<br>• Did you have a situation where you had to see a substitute doctor and how did that work out for you?
3. How important is contact with your specialist to you?<br>• How often do you have appointments or are you in contact by phone?<br>• How did the availability of your specialist change during the pandemic compared to before?<br>• Was there a situation where appointments were postponed for an extended period of time? How did you cope with this situation?
4. Did you have to perform treatments in the hospital since the beginning of the pandemic?<br>• Were the appointments kept or postponed?<br>• Did you have to visit the outpatient clinic for an emergency?<br>• How did you receive treatment?<br>• How did this make you feel?
5. Have you attended telemedicine appointments?<br>• What was your experience with this?<br>• Can you imagine having a conversation with your doctor via video?<br>• What advantages or disadvantages might this have for you?

6. To what extent is it possible for you to carry out your therapies or training sessions regularly during the pandemic compared to the time before?

- What alternatives have you used since the beginning of the pandemic?
- Have you used any online services since the beginning of the pandemic to continue therapies or training sessions (Zoom training)?

7. What regular measures do you take on your own to ensure optimal therapy for yourself?

8. How does the corona pandemic affect your lifestyle?

- To what extent has your physical exercise changed compared to before?
- To what extent has your dietary intake changed compared to before?
- How does the pandemic affect your psychological well-being?

9. What are your greatest fears in the current situation?

10. What is your opinion about the Covid-19 vaccine?

11. What are the biggest challenges for you regarding the pandemic and what are your personal wishes for the future?

Closing: We have reached the end of the interview. Is there anything else you would like to share that was not addressed in this interview?
