# Supplementary material for: COVID-19 triggered a physically active lifestyle of people with cardiovascular diseases: Results of a small Austrian qualitative study
Source: Front Public Health. 2022 Aug 12;10:947250. doi: 10.3389/fpubh.2022.947250 (PMC9417466; doi:10.3389/fpubh.2022.947250)
Supplement: Supplementary file 2 [file Table_2.pdf]

Supplementary Table 2: Codes, sub-codes, descriptions, and sample quotes on the impact of the pandemic on physical activity patterns.

Code and Sub-codes	Description	Sample quote
**Motivation for physical activity**	**Explanations of motivational drivers for engaging in physical activity.**	
Intrinsic Motivation	Expressing feelings of pleasure, enjoyment and satisfaction when talking about physical activity patterns.	"I have to say that I have moved more, quite simply. And that I simply enjoy it, yes. Or the gymnastics in the evening and so on. I look forward to that." [EL11: 233-233]
Integrated Regulation	Emphasizing being a physically active person, claiming to be an "Exerciser".	"And a little bit (…), you can't. Either you do it right or not at all." [EL6: 115-115]
Identified Regulation	Engaging in physical activity because it is important to attain a desired outcome (health or others).	"… still try to do exercise so that the heart muscle is further strengthened." [HB1: 2-2]
Introjected Regulation	Engaging in physical activity to avoid feeling guilty or shamed or to receive acknowledgement.	"Because I don't put myself under any more stress by training". [EL2:117-117]
External regulation	Engaging in physical activity due to an advice of a physician, therapist, family member, friend, etc.	"First of all, they recommended it to me." [JC2: 54]
Amotivation	Expressions indicating that physical activity is not very important to the participant.	"… so I hardly, if you compare it with normal movement patterns, hardly went out and hiked or any other movements." [HB3: 48-48]

**Physical activity patterns**	**Descriptions of physical activity patterns and preferences of study participants before Covid-times.**	
Moderate outdoor exercise	Moderate forms of outdoor exercise: powerwalking, hiking, biking, golf, archery, skiing	"We go hiking a lot ... in summer, we go biking. [EL6: 109-109]
Moderate to vigorous indoor exercise	Moderate to vigorous forms of exercise using indoor sporting facilities: group fitness, exercising in a fitness club, swimming, team sport, aerobics	… before Corona, when I think, I went swimming regularly, I went to the fitness center regularly." [JC3: 58-58]
Moderate to vigorous exercising at home	Moderate to vigorous forms of exercising at home: gymnastics at home, TV-led exercise programs, Cardio-training at home.	"I did all that myself, I put together all the exercises myself. And I also trained before. I did that before …" [HB2: 50-50]
Leisure behavior	Walking and other leisure behavior that includes walking (going shopping, housekeeping, gardening)	"… most important thing is the garden. Because …, I do the whole garden and there is so much movement." [EL1: 47-47]
**Impact of the pandemic on physical activity patterns**	**Descriptions of weather and how the restrictions imposed during lockdowns affected physical activity patterns.**	
Continuation of moderate physical activity with no restrictions	Perceiving no restrictions in physical activity during lockdowns because of being used to do activities that were not affected by the measures: moderate outdoor exercise, exercising at home.	"Yes, I don't go to fitness studios anyway. I … move, outdoors, in nature." [PJ4: 37-37]

Cessation/reduction of moderate to vigorous physical exercise	Perceiving restrictions in moderate to vigorous physical exercise because sporting facilities were closed.	"… they did not open in between … they have renovated the swimming pool and … [I] do not know whether they open it or not …. unfortunately." [JC3: 60-60]
Continuation of leisure behavior with no/minor restrictions	Perceiving minor/no restrictions in walking and other leisure behavior that includes walking because these activities were hardly/not affected by the lockdown measures (going shopping, housekeeping, gardening).	"Walk with the dog, was possible before and after. So, there were no differences at all." [KS1: 48-48]
Cessation/reduction/modification of leisure behavior	Perceiving restrictions in walking and other leisure behavior that includes walking due to lockdown measures (going shopping, going out, housekeeping, gardening).	"… and I've always been outside every day, whether after work somewhere or now on the weekend, but in the first lockdown, well that was just stricter, I say …, you just ordered food or whatever, yes. But otherwise not really." [KS2: 84-84]
**Strategies to maintain physical activity**	**Strategies taken by participants to maintain physical activity during and after lockdowns**	
Integration of walking into daily routine	Indications of participants that daily walking tours has become a routine for them.	And I walked five or six kilometers every day, in one or one and a half hours. And that's actually how it all started." [EL11: 3-3]
Integration of moderate exercising at home into daily routine	Indications of participants that daily walking tours has become a routine for them.	"I regularly got on my exercise bike … and cycled for about half an hour with interruptions." [HB1: 28-28]

Increase in outdoor leisure activities	Indications of participants that they increased frequency and duration of walks or were doing more gardening.	“And just go for a walk a lot. That's my ... we've really been walking, since Corona is now, now the 2nd lockdown, three, four kilometers every day.” [EL1: 46-46]
Increase in moderate outdoor exercise	Indications of participants that they increased frequency and duration of moderate outdoor exercise (walking, hiking, biking).	“But, as I said first, we also cycled a lot. And there was always something. We hiked there, hiked there ...” [EL3: 104-104]
Introduction/increase of moderate to vigorous outdoor exercise	Indications of participants that they increased or entered into moderate to vigorous outdoor exercises.	“My sport now is chopping wood. The meter pieces are really heavy anyway.” [EL2: 34-34]
Replacement indoor exercise by moderate outdoor exercise	Indications of participants that they replaced moderate to vigorous indoor exercise by moderate outdoor exercise (walking, hiking, biking)	“… as we have already discussed, the omission of the indoor activities, we have then compensated in part with hikes.” [KS4: 120-120]
Replacement indoor exercise by moderate to vigorous exercise	Indications of participants that they replaced moderate to vigorous indoor exercise by moderate to vigorous outdoor exercise (work with a personal trainer outdoors, swimming in the lake)	“As then in summer, I just went swimming then … let's say we swam a lot or went swimming more … in [the] lake, yes. [JC3: 62-64]
Resuming physical exercise due to release of measures	Experiences by participants when resuming indoor exercising after measures have been released.	“Yes, we were lucky that the golf course was only closed for a few weeks. It was … usable under certain conditions during the first lockdown.” [KS4: 96-96]

Influencing factors	External factors influencing physical activity patterns mentioned by participants	
Physical Impairment	Indications by study participants that they felt limited in their physical activity due to the burden of disease or other injuries or co-morbidities.	"I go out less since I had the elbow fall. Doesn't really have anything to do with Corona, but I have the fear in my head of falling. As a result, I go out less on my own." [JC1: 68-68]
Time of the year	Indications by study participants that daylight, weather, or temperatures influenced their physical activity patterns.	"But you just had the … spring, had the advantage, they went to the parks, they went for a walk. But now almost no one goes out. What do you want to do? Everything is gloomy. And the weather, if you look at it." [EL2: 13-13]
Availability of time	Indications by study participants that availability of time influenced their physical activity patterns.	"In any case, that has intensified, because now that I'm in home office, it's easier for me to find time. Now from the sports point of view." [EL9: 47-47]
Partner, friends	Indications by study participants that their partner or friends influenced their physical activity patterns.	"My wife anyway, she is constantly on the move. She must do that because of her spine. And I move around, too." [EL6: 109-109]
Availability of garden/proximity to green natural areas	Indications by study participants that the availability of a garden or the proximity to green natural areas influenced their physical activity patterns.	"… and besides I still have my garden in Lower Austria which also occupies me and distracts me and there actually everything does not matter." [KS4: 44-44]
